# Moving the Goalposts in Hypertensive Risk Assessment: The AVI/LVGLS Ratio as a Sensitive Detector of Maladaptive Ventricular–Arterial Coupling

**DOI:** 10.1111/echo.70558

**Published:** 2026-07-11

**Authors:** Giancarlo Trimarchi, Fausto Pizzino, Massimiliano Mariani

**Affiliations:** ^1^ Interdisciplinary Center for Health Sciences Scuola Superiore Sant'Anna Pisa Italy; ^2^ Ospedale Del Cuore “G. Pasquinucci” Fondazione Toscana Gabriele Monasterio Massa Italy

**Keywords:** arterial velocity‐pulse index, ASCVD risk, echocardiography, global longitudinal strain, hypertension, ventricular–arterial coupling

## Abstract

**Purpose:**

This commentary discusses the AVI/LVGLS ratio as an emerging, fully non‐invasive marker of ventricular–arterial coupling (VAC) for refining cardiovascular risk assessment in hypertension beyond conventional indices such as left ventricular ejection fraction and Ea/Ees.

**Methods:**

We critically appraised the study by Zhang et al., which evaluated the association between the arterial velocity‐pulse index to left ventricular global longitudinal strain ratio and estimated 10‐year atherosclerotic cardiovascular disease (ASCVD) risk in hypertensive patients. The commentary integrates these findings with prior evidence on arterial stiffness, myocardial deformation, and VAC assessment.

**Results:**

Zhang et al. showed that AVI/LVGLS differed significantly between hypertensive patients and normotensive controls and was independently associated with elevated estimated 10‐year ASCVD risk. An AVI/LVGLS ratio below the fifth percentile predicted high ASCVD risk after multivariable adjustment, while the composite index demonstrated better discrimination than AVI, LVGLS, or conventional ventricular–vascular index alone. These findings support the incremental value of combining vascular load and myocardial deformation into a single echocardiography‐compatible parameter.

**Conclusion:**

AVI/LVGLS may provide a practical bridge between vascular and myocardial phenotyping in hypertension, offering a sensitive marker of maladaptive VAC and subclinical cardiovascular risk. Prospective, multicenter validation is required before implementation in routine risk stratification or therapeutic monitoring.

## Graphical abstract

Graphical Abstract Text: The AVI/LVGLS ratio—combining arterial velocity‐pulse index (oscillometric cuff) and left ventricular global longitudinal strain (2D speckle‐tracking)—as a non‐invasive ventricular–arterial coupling marker for 10‐year ASCVD risk stratification in hypertension. AVI/LVGLS <fifth percentile: OR = 6.11 (95% CI: 1.35–27.6); AUC = 0.726versus. VVI 0.554. *Abbreviations*: AVI: arterial velocity‐pulse index; ASCVD: atherosclerotic cardiovascular disease; AUC: area under the curve; Ea/Ees: arterial‐to‐end‐systolic elastance ratio; LVGLS: left ventricular global longitudinal strain; OR: odds ratio; VAC: ventricular–arterial coupling; VVI: ventricular–vascular index.

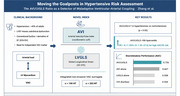

**Abbreviations**
ASCVD: Atherosclerotic Cardiovascular Disease
AUC: Area Under the Curve
AVI: Arterial Velocity‐Pulse Index
cfPWV: Carotid‐Femoral Pulse Wave Velocity
CI: Confidence Interval
E/e′: Ratio of Early Mitral Inflow Velocity to Mitral Annular Early Diastolic Velocity
Ea: Arterial Elastance
Ees: End‐Systolic Elastance
EF: Ejection Fraction
ESV: End‐Systolic Volume
FABP4: Fatty Acid‐Binding Protein 4
GLS: Global Longitudinal Strain
HDL‐C: High‐Density Lipoprotein Cholesterol
HFpEF: Heart Failure with Preserved Ejection Fraction
IL‐6: Interleukin‐6
LASSO: Least Absolute Shrinkage and Selection Operator
LV: Left Ventricle
LVEF: Left Ventricular Ejection Fraction
LVGLS: Left Ventricular Global Longitudinal Strain
LVMI: Left Ventricular Mass Index
MMP‐1: Matrix Metalloproteinase‐1
MMP‐2: Matrix Metalloproteinase‐2
NT‐proBNP: N‐Terminal Pro‐B‐Type Natriuretic Peptide
OR: Odds Ratio
PWV: Pulse Wave Velocity
SGLT‐2: Sodium‐Glucose Cotransporter‐2
SV: Stroke Volume
VAC: Ventricular–Arterial Coupling
1 VVI: Ventricular–Vascular Index

## 1 Main Text

Hypertension remains one of the most prevalent modifiable cardiovascular risk factors globally, affecting nearly 45% of adults and substantially contributing to atherosclerotic cardiovascular disease (ASCVD) burden and premature mortality [1]. Despite the availability of validated risk stratification models such as the China‐PAR framework [2], early identification of individuals at elevated 10‐year ASCVD risk remains challenging, particularly because conventional echocardiographic parameters—including left ventricular ejection fraction (LVEF)—often fail to detect subclinical cardiovascular dysfunction in hypertensive patients with preserved systolic function. In this context, ventricular–arterial coupling (VAC), defined as the hemodynamic interaction between the left ventricle and the arterial system, has garnered increasing interest as an integrated marker of cardiovascular performance and risk. Traditionally assessed through invasive pressure–volume loop analysis or echocardiographic estimates of the arterial‐to‐ventricular elastance ratio (Ea/Ees), VAC assessment has been hampered by methodological limitations in clinical settings. The article by Zhang et al. published in this issue proposes a novel, fully non‐invasive approach to quantifying VAC—the ratio of the arterial velocity pulse index to left ventricular global longitudinal strain (AVI/LVGLS)—and examines its independent association with estimated 10‐year ASCVD risk in hypertensive patients [3, 4].

The conventional echocardiographic Ea/Ees ratio, while conceptually grounded in cardiac energetics, carries a fundamental limitation: when computed via the simplified formula (Ea/Ees ≈ ESV/SV = [1−EF]/EF), it reduces to a mathematical derivative of LVEF, offering limited additional information beyond ejection fraction [4]. This circularity has prompted the search for VAC surrogates that independently assess arterial and myocardial function. In recent years, the ratio of pulse wave velocity (PWV) to global longitudinal strain (GLS) has emerged as a more informative marker, demonstrating superior correlation with subclinical target organ damage and adverse outcomes compared with Ea/Ees [5, 6], though cfPWV measurement requires dedicated equipment and trained operators that are not universally available. The arterial velocity pulse index (AVI), derived from non‐invasive oscillometric cuff measurements, and provides a low‐cost, operator‐independent surrogate of arterial stiffness [7]. Left ventricular GLS (LVGLS), assessed by two‐dimensional speckle tracking echocardiography, is one of the most sensitive markers of subclinical left ventricular dysfunction, with independent prognostic value across multiple cardiovascular conditions [8, 9]. By combining these two complementary indices, the AVI/LVGLS ratio integrates arterial load and myocardial contractile performance into a single dimensionless parameter, offering a practical surrogate of maladaptive ventricular–arterial interaction. This approach was formally proposed by Wu et al. [10], and first validated in a hypertensive population by Yang et al. [11]., who demonstrated that the AVI/GLS ratio was significantly more impaired in hypertensive versus normotensive individuals (−0.77 ± 0.29 vs. −0.66 ± 0.28, *ρ* = 0.001) and outperformed traditional Ea/Ees in discriminating cardiovascular changes associated with hypertension (AUC = 0.645), the current work by Zhang et al. [3], extend this validation to risk stratification, shifting the focus from the diagnosis of hypertension per se to cardiovascular risk quantification.

In a single‐center cross‐sectional study enrolling 190 hypertensive patients and 203 age‐ and sex‐matched normotensive controls, Zhang et al. [3], demonstrated that, compared with normotensive controls, hypertensive patients exhibited significantly higher AVI/LVGLS values (*p* < 0.05), consistent with greater arterial load relative to myocardial contractile function. Strikingly, within the overall population, AVI/LVGLS showed an inverse correlation with estimated ASCVD risk—subjects in the high‐risk category (10‐year ASCVD risk ≥10%) displayed lower AVI/LVGLS values than low‐risk counterparts. This inverse relationship, partly driven by age‐related impairment of myocardial deformation in older high‐risk individuals, underscores the nonlinear hemodynamic trajectory of ventricular–arterial uncoupling across the continuum of cardiovascular risk. In multivariable logistic regression adjusting for age, sex, hypertension status, HDL‐C, and left ventricular mass index (LVMI), an AVI/LVGLS ratio below the fifth percentile was independently associated with elevated estimated 10‐year ASCVD risk (OR = 6.11, 95% CI: 1.35–27.6, *p* = 0.019). LASSO regression further confirmed AVI/LVGLS as one of six key predictors of high ASCVD risk. The discriminatory performance of the composite index (AUC = 0.726) was markedly superior to that of the ventricular–vascular index (VVI, defined as Ea/Ees; AUC = 0.554), AVI alone (AUC = 0.667), and LVGLS alone (AUC = 0.558), underscoring the additive value of integrated ventricular–arterial assessment over individual components [3].

These findings build on growing evidence that myocardial deformation parameters correlate with ASCVD risk in hypertension. Chen et al. [12], previously demonstrated, using the same China‐PAR framework and a similar hypertensive population, that layer‐specific GLS is independently associated with high 10‐year ASCVD risk: specifically, whole‐wall GLS and mid‐myocardial GLS emerged as independent predictors after full multivariate adjustment, the present study by Zhang et al. [3], now shows that incorporating the arterial stiffness component through AVI substantially enhances this risk‐predictive framework. This conceptual evolution mirrors the paradigm established for the PWV/GLS ratio, in which the integration of arterial and myocardial dimensions of VAC provides more comprehensive cardiovascular risk phenotyping than either parameter alone [13].

Importantly, the study identifies independent determinants of AVI/LVGLS, with age, systolic blood pressure, and E/e′ ratio negatively associated and LVEF positively associated with the index. This pattern is consistent with the well‐documented age‐related deterioration of VAC—driven by progressive arterial stiffening and declining longitudinal myocardial deformation—and with the hemodynamic effects of pressure overload on diastolic function [5]. The inverse relationship with E/e′ further suggests that AVI/LVGLS captures aspects of both systolic and diastolic ventricular–arterial interaction in hypertension.

The mechanistic substrate underlying impaired VAC in hypertension is well established. Chronic pressure overload promotes arterial stiffening through collagen deposition, elastin fragmentation, and endothelial dysfunction, accelerating pulse wave velocity and shifting wave reflections into systole. This increases left ventricular afterload, impairs coronary diastolic perfusion, and drives myocardial fibrosis and hypertrophy—ultimately reducing longitudinal deformation and worsening GLS. The bidirectional deterioration of both AVI and LVGLS in sustained hypertension renders their ratio a sensitive detector of progressive ventricular–arterial uncoupling [4]. At the molecular level, recent data from the STANISLAS cohort demonstrate that impaired VAC (assessed by PWV/GLS) is associated with elevated circulating biomarkers of inflammation and extracellular matrix remodeling, including IL‐6, FABP4, and MMP‐1, while MMP‐2 and NT‐proBNP showed an inverse association with VAC deterioration in a large community‐based population [13], suggesting that AVI/LVGLS may not only reflect hemodynamic dysfunction but also serve as a surrogate of systemic biological aging relevant to atherosclerosis progression.

Several limitations of the study by Zhang et al. [3] deserve consideration. First, the cross‐sectional design precludes causal inference and restricts the primary outcome to estimate—rather than observed—cardiovascular events. While the China‐PAR model is a validated tool for 10‐year ASCVD risk prediction in the Chinese population [2], risk estimates are inherently subject to model‐specific calibration uncertainties. Whether AVI/LVGLS provides independent prognostic value beyond China‐PAR‐estimated risk in hard‐event prediction remains to be established in prospective longitudinal cohorts.

Second, the study was conducted at a single center in a Chinese population, and the external validity of the proposed cutoff (fifth percentile) requires multicenter validation across different ethnic groups and hypertension phenotypes. Careful attention to measurement standardization will be essential, as both AVI and speckle‐tracking GLS are susceptible to technical variability related to device‐specific algorithms, body habitus, and image quality, the overall discriminatory performance of AVI/LVGLS, while superior to individual components, remained moderate (sensitivity 74.5%, specificity 55.2%), with the optimal cutoff defined empirically within the study cohort—a threshold that requires prospective external validation before translation into clinical decision‐making algorithms.

Third, the study population excluded patients with significant structural heart disease, atrial fibrillation, and heart failure, which are conditions in which VAC assessment is increasingly relevant [4, 5]. Future studies should explore AVI/LVGLS across the broader hypertensive phenotypic spectrum, including those with diastolic dysfunction, left ventricular hypertrophy, and early heart failure with preserved ejection fraction. Critically, longitudinal evidence is needed to establish whether pharmacological interventions aimed at reducing arterial stiffness or improving LVGLS—such as renin–angiotensin system blockade or sodium–glucose cotransporter‐2 inhibitors—translate into measurable AVI/LVGLS improvement and downstream cardiovascular risk reduction.

In conclusion, the work by Zhang et al. [3] provides clinically relevant evidence supporting the AVI/LVGLS ratio as a non‐invasive, fully echocardiography‐compatible VAC marker that independently stratifies 10‐year ASCVD risk in hypertensive patients. By coupling a widely available arterial stiffness index with the proven prognostic sensitivity of GLS, this parameter offers an accessible bridge between vascular and myocardial phenotyping—complementing rather than supplanting established risk models. Moving beyond LVEF, the integration of ventricular–arterial assessment into routine echocardiographic practice holds tangible promise as a cornerstone of imaging‐guided cardiovascular prevention [9, 14].

## Ethics Statement

The authors have nothing to report.

## Conflicts of Interest

The authors declare no conflicts of interest.
